# Human Induced Pluripotent Stem Cells and the Modelling of Alzheimer’s Disease: The Human Brain Outside the Dish

**DOI:** 10.2174/1874205X01711010027

**Published:** 2017-09-30

**Authors:** Godwin Tong, Pablo Izquierdo, Rana Arham Raashid

**Affiliations:** 1 College of Medical and Dental Sciences, University of Birmingham, Edgbaston, Birmingham, B15 2TT, United Kingdom; 2 Department of Neuroscience, Physiology and Pharmacology, University College London, Gower Street, London, WC1E 6BT, United Kingdom

**Keywords:** Alzheimer’s Disease, Induced Pluripotent Stem Cells, AD Modelling, Human-mouse chimeric model, Microglia, Neuronal grafts, Neuroinflammation

## Abstract

**Background:**

Neurodegenerative diseases like Alzheimer’s Disease (AD) are a global health issue primarily in the elderly. Although AD has been investigated using primary cultures, animal models and post-mortem human brain tissues, there are currently no effective treatments.

**Summary:**

With the advent of induced pluripotent stem cells (iPSCs) reprogrammed from fully differentiated adult cells such as skin fibroblasts, newer opportunities have arisen to study the pathophysiology of many diseases in more depth. It is envisioned that iPSCs could be used as a powerful tool for neurodegenerative disease modelling and eventually be an unlimited source for cell replacement therapy. This paper provides an overview of; the contribution of iPSCs towards modeling and understanding AD pathogenesis, the novel human/mouse chimeric models in elucidating current AD pathogenesis hypotheses, the possible use of iPSCs in drug screening, and perspectives on possible future directions.

**Key messages:**

Human/mouse chimeric models using iPSCs to study AD offer much promise in better replicating AD pathology and can be further exploited to elucidate disease pathogenesis with regards to the neuroinflammation hypothesis of AD.

## 1 INTRODUCTION

Alzheimer’s Disease (AD) is the most common type of neurodegenerative disease, affecting about 30 million people worldwide and almost a million people in the UK [1]. The prevalence rates of AD-related dementia also rise substantially with age particularly between 65-80 years [2]. It has an estimated economic burden of £26 billion a year in the UK but still lacks a cure [3]. There are two main forms of AD: familial AD (FAD) and sporadic AD (SAD). FAD and SAD are largely similar clinically, both characterised by progressive cognitive decline that results in the impairment of forming memories, planning, problem solving, visuospatial skills and orientation. These deficits pose a major barrier to basic functions of daily life especially in the old age and can result in premature death as well [4]. Histopathologically, both subtypes are associated with accumulation of extracellular senile plaques consisting of amyloid-β peptides (Aβ) and neurofibrillary tangles formed by hyperphosphorylated tau protein, resulting in axonal transport defects as well as loss of neurons and synapses in the cerebral cortex and certain subcortical regions [5-7]. This loss results in gross atrophy of affected regions, such as the hippocampus, entorhinal cortex, temporal lobe, parietal lobe, frontal cortex and cingulate gyrus [8]. However, whether amyloid plaques and tau pathology are a cause or consequence of AD and the relation between both, is still unclear in humans [9].

FAD has an early onset (usually before the age of 65), familial element and accounts for <5% of all AD cases. It is primarily due to rare autosomal dominant mutations in the amyloid precursor protein (*APP*) gene and in the presenilin genes, *PSEN1* and *PSEN2*. APP is an integral membrane protein found in neural synapses and is the precursor molecule for Aβ production through proteolysis [10]. Both PSEN1 and PSEN2 are required in the formation of a γ-secretase complex which functions to cleave APP, leading to Aβ formation [11]. On the other hand, SAD has a late onset (after age 65) and sporadic character, accounting for >95% of all AD cases. However, most ideas about disease pathogenesis have been derived from FAD models [12]. Despite having a strong heritable element, genome-wide association studies (GWAS) on SAD models have only reported 21 risk loci of varying consistency, with apolipoprotein isoform E4 (APOE4) and apolipoprotein J being most consistent [13, 14]. It should be noted that *APOE* is significantly expressed in microglia as are a few of the other loci (*CR1, CD33 and TREM2*) from the GWAS data, which are also ascribed to the innate immune system. This suggests the potential importance of the immune system and possibly a role for microglia in AD [15, 16]. Although the other hits have lesser effect on AD risk as compared to *APOE*, the GWAS data does highlight the multifactorial element of SAD and has provided us with more information on the pathophysiological pathways of AD.

## 2 USE OF iPSC TECHNOLOGY IN NEUROLOGICAL DISEASE MODELLING

Pluripotent stem cells have an immense utility in studying the molecular basis of many diseases as they can differentiate into any cell type. Takahashi and Yamanaka pioneered the generation of pluripotent stem cells in humans by inducing them from fibroblasts [17]. These induced pluripotent stem cells (iPSCs) can be reprogrammed from fibroblasts by retroviral transduction and expression of four transcription factors OCT4, SOX2, KLF4, and c-MYC or OCT4, SOX2, NANOG, and LIN28 [17, 18]. Since their discovery, the field of iPSCs has exploded and this technology has opened opportunities for modelling human diseases, enabling the study of pathogenesis at a molecular level for many neurological diseases including AD in *vitro and in vivo*. For more information on the progress so far one can refer to the reviews by Ross and Akimov, Dolmetsch and Geschwind as well as Jung *et al.* [19-21] Currently, pathogenesis studies require the use of post-mortem tissues and transgenic animal models [22]. However, the former can be difficult to obtain and might not reflect early stage changes. On the other hand, murine models have been used extensively but they do not reflect the human neural phenotype, primarily due to various anatomical differences in the structure and development between human and rodent brains [23]. For example, the human subventricular zone is more developed than that of rodents and this might affect the progression of dementia [24]. It is also suggested that underlying microscopic differences between humans and rodents could affect the validity of current preclinical models, hence possibly explaining the slow translation from bench to bedside [25]. One advantage of using iPSCs is that they can be generated from elderly patients which is beneficial for the study of late onset neurodegenerative diseases such as AD and Parkinson’s Disease (PD). Furthermore, since human iPSCs are genetically specific to patients, they enable us to study the effect of human genetic background on neurological diseases which is difficult to replicate in murine models.

It is a difficult task to produce models that accurately reflect neurological disease and this is particularly true for AD modelling which unlike the other neurological diseases, is less well characterised in terms of mimicking disease phenotypes. Because AD is a disease of the central nervous system, obtaining patients’ tissue before death is challenging. Furthermore, mutations introduced into mouse genes do not recapitulate all human. AD pathology thus making transgenic mice models less predictable [26]. As such, most existing iPSC models are *in vitro* [20]. Recently, a number of SAD patient derived iPSC cell lines have been created in an attempt to characterise SAD pathogenesis with *APOE* consistently having differential expression amongst a myriad of target genes as mentioned earlier [27-29]. The lack of a patterned genetic lesion for SAD has proven to be a challenge to model SAD. As such, the *in vitro* models of AD mentioned here revolve around FAD utilising iPSCs.

This paper will describe; (1) the recent progress of the use of iPSCs for *in vitro* and *in vivo* modelling of AD, (2) a promising novel model of iPSC-derived neuronal transplant, and (3) discuss perspectives on its possible future directions.

### 2.1 *In Vitro* iPSC Models of AD

#### 2.1.1 *PSEN* Models of FAD

iPSCs are derived from patients with specific point mutations in *PSEN1*, *PSEN2* or *APP* genes based on the rationale that downstream actions of the initial mutations are similar. To examine whether these models reflect AD pathology, Aβ40, Aβ42 and Aβ42/40 peptide ratios are often used as markers. The prevailing ‘amyloid cascade hypothesis’ of AD pathogenesis maintains that elevated plasma Aβ40/42 ratio is associated with the disease. However, no unequivocal evidence exists to show whether this phenomenon is due to a toxic gain of function by overproduction of Aβ42, or a partial loss-of-function mechanism leading to decreased generation of Aβ40 [30]. There are *in vitro* models with *PSEN1* and *PSEN2* mutations showing elevated Aβ42 secretion, consistent with the pathogenesis views [31-33]. It is true, however that overall gene expression patterns are not always drastically different between mutant and control lines unless examined in detail [33]. In other *PSEN1* mutant models, such as the L166P mutation which is known to cause an aggressive form of FAD, the Aβ42/40 ratio was higher after overexpressing *PSEN1* but this was due to a large decrease in Aβ40 peptides in the L166P neurons [34]. Another *PSEN1* variant D835N model involving a loss of function mutation showed decreased secretion of both Aβ peptides, confirming the successful suppression of endogenous γ-secretase activity [34]. Hence there is a growing notion suggesting Aβ40 might decrease plaque formation and therefore increase the neurotoxicity of Aβ42 peptides produced by PSEN1/2 FAD mutants [35].

#### 2.1.2 *APP* Models of FAD

FAD can also be caused by duplication of the *APP* gene on chromosome 21 [36]. Models of *APP* mutants have also been created [12, 37]. One model developed by Muratore *et al*. differentiated neurons derived from iPSCs from patients with V717I mutation affecting *APP* expression. They showed an increase in levels of total and phosphorylated tau, along with an increase in β-secretase cleavage of APP resulting in increased levels of both APP secretase and Aβ. They also demonstrated that by treating the cells with specific antibodies, total tau and Aβ levels were reduced [37]. Israel *et al*. [12] also attempted to model both FAD and SAD phenotypes by showing elevated levels of Aβ40 and tau phosphorylation in the iPSC-derived neurons of FAD and SAD patients when compared with neurons from non-demented age-matched individuals. Active GSK3β, a kinase involved in the phosphorylation of tau was observed to be elevated as well, leading to increased tau phosphorylation. This study extended phenotypic characterisation by looking at endosomal and synaptic markers since AD severity is known to be associated with synaptic loss. *APP* mutants showed increased Rab5+ endosomes, a shared phenotype with SAD, but interestingly, there were no differences in the synaptic marker synapsin-1 in mutants, contrasting with other studies showing a reduction of synapsin-1 [38]. Kondo *et al*. [39] compared 7 types of *APP* mutations including *APP* E693 deletion and *APP* V717L mutation but the cell lines did not consistently replicate the same phenotypes possibly due to varying differentiation periods. Moreover, the E693Δ deletion has been shown to be antiamyloidogenic and neither extracellular amyloid plaques nor tau tangles are formed in transgenic mice expressing *APP* E693Δ, suggesting that not all currently available models are successful in exhibiting AD-like pathology, and so not all are valid for the generation of AD iPSC lines [40, 41]. Additionally there are discrepancies in the culture time across these studies. Similar to *in vivo* brains, *in vitro* neurogenesis requires extended culture periods (months) to observe mature features of action potential patterns and synaptic activity [42, 43]. Furthermore, with respect to tau pathology, adult tau isoform expression is low even after 90 days of differentiation of certain iPSC cell lines with *MAPT* mutation [44, 45]. This raises concerns regarding the validity of earlier models with culture periods of weeks. Taken together, these studies reflect the inherent variability of iPSCs and highlight the need for further refining of FAD *in vitro* models. In particular, more studies regarding neuronal culture periods should be conducted to determine the optimal time frame to observe the critical phenotypes of neuro-ageing such as axonal degeneration which is rarely observed. iPSC-derived neurons could also be further characterised to ensure the neuronal identity generated is similar to that of known AD-affected regions such as the cholinergic CA1 region of the hippocampus, by comparing gene expression profiles to that of known RNA sequencing databases [46, 47].

In 2012, a seminal study conducted by Shi *et al*. [48] used a different approach whereby iPSCs derived from patients with Down’s Syndrome were used to generate cortical cholinergic neurons over a differentiation period of 28-100 days. Since Down’s Syndrome patients have a predilection to develop AD, they hypothesised that iPSC-derived cortical neurons taken from Down’s Syndrome patients could be used to model early onset FAD as they harboured three copies of *APP*. It has also been shown that increased *APP* expression in mice and humans results in amyloid plaques, early onset dementia and other neuropathological hallmarks [36, 49]. Their cell lines revealed production of neuronal Aβ secretion, plaque formation and altered tau protein localisation and phosphorylation. More importantly, they showed that this phenotype could be observed within months instead of years and are free from spontaneous mutations introduced by cellular reprogramming.

Although this review focuses on neuronal pathology and lines where amyloid metabolism is affected, it needs to be acknowledged that other iPSC lines have been generated. For example, iPSC-derived astrocytes have been obtained from skin fibroblasts of non-demented *APOE* ε4 carriers (*APOE* is predominantly expressed by astrocytes in the brain, as well as microglia [16]. Interestingly, not only do these astrocytes show an increased apoE lipoprotein secretion but they also show impaired neurotrophic support when co-cultured with iPSC-derived neurons, as compared to astrocytes derived from *APOE* ε3 carriers [50]. Given differences in gene expression between human and rodent astrocytes, this approach is of particular relevance to model human AD. More recently, newer three-dimensional (3D) neural cell culture models have been developed in an attempt to link together the multiple putative hypotheses of AD pathogenesis [51-53]. These 3D models show an improvement in being able to explore the impact of both amyloid beta pathologies and taupathies in a single model compared to their 2D counterparts [51]. Additionally, the 3D environment is better at replicating clinically-observed AD pathology (i.e. higher Aβ42 levels) compared to the 2D models [52], which means that the 3D-modeled pathology is similar to that observed in the in vivo iPSC models or clinical cases of AD. These advanced 3D neuronal models incorporating human iPSCs hold promise in elucidating less well-known constructs of AD pathogenesis in the future.

#### 2.1.3 In Vivo iPSC Models of AD

Work in transgenic mice have undoubtedly advanced the understanding of AD significantly, especially by providing evidence to support the amyloid cascade hypothesis. However, despite efforts to develop sophisticated transgenic mice models resembling AD in terms of amyloid deposition, none of them are able to truly replicate the entire spectrum of biochemical, cellular and behavioural pathology of AD patients [54]. Currently, transgenic mice carry mutations in either *APP*, *PSEN1/2*, or tau (*MAPT*) genes but as discussed, the substantial amyloid deposition that occurs in SAD is not due to any one particular mutation. Also, Aβ and tau accumulation are not exclusive to AD and exist in other neurodegenerative disorders and even in elderly non-demented individuals [55]. Thirdly, AD patients who were vaccinated against Aβ42 showed significant reduction of amyloid plaques within the grey matter, without a concomitant rescindment of dementia [56]. Taken together, these limitations show that accumulation of Aβ in the brain alone is not sufficient to model human AD, thus casting some doubt on the amyloid cascade hypothesis as a critical pathogenic factor in SAD [54].

#### 2.1.4 Chimeric Models of AD

Attempts to address these problems have been made via the transplantation of patient-specific iPSC-derived neurons into the brains of AD affected mice. Such human iPSC/mouse chimeric models allow modelling of patient-specific neurodegenerative diseases in a relatively physiological, three-dimensional environment. The healthy mouse brain provides the scaffold for neuronal re-innervation and stabilisation of connectivity, allowing the study of axonal plasticity of healthy and diseased human iPSC-derived neurons - a phenotype less characterised in neurodegenerative diseases like AD. Furthermore, chimeric models have the potential to generate more relevant *in vivo* neuropathological data to perform drug validation studies. Initially it was thought that integration into the host brain would be a problem but many studies have now suggested that human iPSCs-derived neurons transplanted into a host brain are capable of surviving from months up to years post-transplantation [57-61]. More importantly, they exhibit synaptic integration with host circuits as seen from patch-clamping experiments displaying spontaneous excitatory postsynaptic currents. It is interesting to note that chimeric models have revealed crucial information about neuronal maturation kinetics whereby it is suggested that neurons mature along their own, species-specific ‘clock’. This is seen from the different pace of axonal maturation where cortical cells differentiated from mouse iPSCs took weeks to integrate whereas human iPSC-derived cells only developed functional synapses after several months [42, 62-64].

Indeed transplant studies are not new and have been studied in other neurodegenerative disease models like PD, opening up a new window for personalised therapeutic options with promising behavioural modifications in preliminary animal studies [58, 59, 65, 66]. Surprisingly despite their potential, the application of such chimeric models to study AD pathogenesis has yet to receive the same attention. Recently, Espuny-Camacho *et al*. [67] created a novel chimeric model for AD, demonstrating human-specific pathological features. They transplanted neuronal precursors differentiated from patient-derived iPSCs into a known FAD mouse model [68] with both *APP* and *PSEN1* mutations. The characterisation of their model has highlighted its relevance for human AD. After differentiating human iPSC-derived cortical precursor cells into mature cortical neurons which were mainly glutamatergic, they used immunogold labelling to show the presence of Aβ deposits nesting within human neuron clusters. They also showed dystrophic neurites surrounding Aβ plaques in the AD chimeric mouse models but not in healthy chimeric mouse controls. Moreover, synaptic properties were also altered as compared to their controls using mouse neuronal clusters grafted in the AD mice. There was an accumulation of the presynaptic markers synaptophysin and glutamate transporter 1 around Aβ plaques amongst human clusters in AD mice. Reduction of dendritic marker MAP2 and postsynaptic marker Homer1 were also observed. Importantly, they demonstrated neuronal degeneration, a phenotype rarely recreated in current models. Using ultrastructural analysis, they showed almost a 50% reduction in neuronal density of human neurons in AD mice six months after transplantation as compared to control grafts using mouse host tissue neurons. Interestingly, this phenotype mimics the time-dependent properties seen in humans as well, with degeneration only commencing months later, providing further evidence for the species-specific clock hypothesis. Interestingly, 33% of human neurons displayed necrosis but not apoptosis and this was absent in human neurons grafted into healthy mouse controls. This suggests that necrotic mechanisms may contribute to neurodegeneration in humans. Lastly, they used genome-wide microarrays to analyse the effects of Aβ exposure on human gene expression. In particular, there was upregulation of genes involved in myelination and downregulation of genes related to memory and cognition, synaptic transmission, and axonal projection. Comparison of their gene expression data with existing human AD data showed a match of 22 out of 37 gene modules, further supporting the accuracy of this model in replicating human AD phenotype. Taken together, this is the first study to generate a chimeric AD model using human iPSC-derived neurons and it highlights the importance of having the appropriate human and molecular background in order to faithfully recapitulate AD phenotypes.

## 3 LIMITATIONS AND FUTURE PERSPECTIVES

While the use of iPSCs in modelling the progression of AD is an exciting prospect, it is not without its limitations [69], both ethical and scientific. The ethical considerations of iPSC use are similar to those that are associated with the use of embryonic stem cells, where iPSCs have the potential to induce formation of gametes that can eventually be crossed in a laboratory setting to set a precedent for cloning individuals in the future. Also, human iPSCs have the potential to be introduced into embryos from different species making it possible to create diseased/healthy chimeric organisms, and it is unclear whether such use is completely ethical. Scientifically, the research implicating iPSC use in cell differentiation and therapy is still in its infancy. For instance, compared to embryonic cells, iPSCs differentiate to other cell types at significantly slower rate and have a higher chance of cell death [70]. There are also concerns of introducing harmful mutations during the process of inducing the formation of iPSCs from adult cells. It is thought that such mutations arise from the retroviruses that are used to generate the iPSCs. Additionally, there is a possibility of integrating viral genome in the human iPSCs which could prove harmful by jeopardizing human genomic stability. This limitation can be offset by employing the use of novel genome editing techniques such as the clustered regularly interspaced short palindromic repeats (CRISPR) associated protein 9 (Cas9) nuclease system to minimise the risk of introducing and propagating harmful mutations [71].

The use of zinc finger nuclease, transcription activator-like factor nucleases (TALENs) or CRISPR/Cas9 technology allows one to genetically correct mutations or introduce mutations and has been used in PD studies where isogenic lines were created by correcting point mutations from patient derived iPSC and PD mutations were induced in human embryonic stem cells [72]. Isogenic studies have also been conducted for other neurological conditions such as Amyotrophic Lateral Sclerosis and Rett’s syndrome but less so for AD [73-75]. Such techniques when applied to AD, make it the ideal control set up and the resulting isogenic cells can reveal more information on the exact effects of a *PSEN/APP* mutation within the same genetic background whilst under endogenous regulation of expression. To date very few studies have attempted to characterise isogenic AD cell lines and as such there is a demand for such further studies [76, 77]. One recent study that explored the use of CRISPR/Cas9 in introducing mutations to study AD found that using this improved genomic editing technique they accurately and efficiently generated both homozygous and heterozygous dominant AD-causing mutations [78].

Additionally, it is long known that microglia play a prominent role in disease pathogenesis in diseases like PD and age-related macular degeneration [79-81]. With respect to AD, microglia have recently gained interest and are thought to be involved in pathogenesis but a unifying hypothesis on the importance of neuroinflammation has yet to emerge [15, 82]. Microglia can adopt several functional states in the healthy brain, with roles in basal surveillance of toxic substances, phagocytosis, synaptic pruning or neuromodulation [83]. During the course of AD, microglia may initially constitute a protective barrier that degrades plaques [84, 85]. However, pro-inflammatory activation of microglia, for instance as a result of the accumulation of misfolded proteins [86] hinders their phagocytic ability and promotes the production and secretion of tissue-damaging cytokines, which are increased both in AD mouse models and patients [87, 88]. Interestingly, blockade of microglial proliferation, which is increased in AD, improves memory function in mice suggesting an overall harmful contribution of these cells in AD [89]. However, current research on the ‘neuroinflammation hypothesis’ of AD utilise murine models where immune responses do not always mimic those of humans, making accurate comparisons difficult [87, 90]. One possible explanation for this could be the differential gene expression, for instance of *TREM2* which is a global regulator of microglial function and is upregulated in AD mice, thus potentially offering some form of neuroprotection [92]. As such, chimeric models might be useful in elucidating more answers in this regard.

Another question that can be posed to transplantation models is the gender difference within AD. Although the prevalence of AD is higher in women, there is an earlier and more severe cognitive decline in men [91]. Many hypotheses have been formulated in an attempt to explain these differences such as hormonal and brain metabolism differences [92]. Interestingly there has been increasing focus on the sexual dimorphism of microglia phenotype in several known AD target brain areas like the cortex and cerebellum [93-96]. Indeed one hypothesis albeit less examined is the possible involvement of microglia via the “microglia dysfunction hypothesis” [97]. Thus, chimeric graft models could be utilised by grafting iPSC-derived microglia into developing AD mice models and explore the effects it has on disease progression. It might be worthwhile including endogenous microglia and inflammation status as an end-point phenotype in future chimeric models while factoring in sex to further characterise the influence of sex and its potential contribution to neuroinflammation in AD.

## CONCLUSION

At a glance, it is clear that chimeric models have important applications in terms of drug screening and therapy. Currently, only four pharmacological agents have been approved for clinical AD treatment by the National Institute for Health and Care Excellence. These cholinesterase inhibitors or NMDA receptor antagonists have very limited, if not null effect on the pathological characteristics of AD and only provide symptomatic relief at best [98]. The average cost of drug development in the UK is estimated to be £1.15 billion with the majority of candidates failing at later stages of clinical trials [99]. Therefore, since patient-derived iPSC neuronal grafts can better replicate neurological disease phenotypes, they might be able to provide a platform for more accurate drug toxicity tests especially when paired with high-throughput screening. Thousands of compounds could be screened against currently considered end-points like Aβ peptides and phosphorylated tau levels or even the elusive neuronal degeneration phenotype. The more reliable results provided could therefore reduce the cost and streamline the process of validating drug targets.

In terms of therapeutics, iPSCs-based regenerative medicine for AD can bypass immune rejection and such therapies potentially revolve around gene correction and iPSCs-derived neuronal transplantation [100]. Although the *in vivo* applications for cell transplantation and endogenous reprogramming for AD are still at early stages of development, their value has been observed *in vitro* and *in vivo* in other neurological diseases including less common tauopathies and past decade [65, 101,102]. As such it is an exciting future ahead for personalised medicine with the advent of more realistic chimeric AD models such as that of Espuny-Camacho *et al*. [67] and others to come [61].

From an overall perspective, although the application of iPSCs to AD is still an emerging field, a number of studies in the past ten years have yielded valuable information on AD pathogenesis. Models to study FAD/SAD are continuously being developed and improved upon as seen from the emergence of transplanted neuronal graft models. The potential of iPSCs to treat patients with AD is enormous but not without difficulties. The challenge now is to use such models while building on previous work to study the interplay between Aβ, tau pathology, neuroinflammation and neurodegeneration so as to better replicate AD phenotype with the long-term goal of achieving the promise of personalised medicine for this devastating disease.

## LIST OF ABBREVIATIONS

AD: = Alzheimer’s Disease
APP: = Amyloid Precursor Protein
Aβ: = Amyloid-β peptides
APO: = Apolipoprotein
FAD: = Familial Alzheimer’s Disease
GWAS: = Genome-Wide Association Studies
iPSC: = Induced Pluripotent Stem Cells
SAD: = Sporadic Alzheimer’s Disease
PD: = Parkinson’s Disease
